# Sub-national analysis and determinants of numbers of antenatal care contacts in Nigeria: assessing the compliance with the WHO recommended standard guidelines

**DOI:** 10.1186/s12884-021-03837-y

**Published:** 2021-05-25

**Authors:** Adeniyi Francis Fagbamigbe, Olugbenga Olaseinde, Vincent Setlhare

**Affiliations:** 1 Department of Epidemiology and Medical Statistics, College of Medicine, University of Ibadan, Ibadan, Nigeria; 2 Health Data Science Group, Division of Population and Behavioural Sciences, School of Medicine, University of St Andrews, St Andrews, United Kingdom; 3 Department of Sociology, Adekunle Ajasin University, Akungba Akoko, Nigeria; 4 Department of Family Medicine and Public Health, Faculty of Medicine, University of Botswana, Gaborone, Botswana

**Keywords:** Antenatal care, Nigeria, WHO guidelines, ANC contacts, Education

## Abstract

**Background:**

Nigeria has unimpressive maternal and child health indicators. Compliance with the WHO guidelines on the minimum number of antenatal care (ANC) contacts could improve these indicators. We assessed the compliance with WHO recommended standards on ANC contacts in Nigeria and identify the associated factors.

**Methods:**

Nationally representative cross-sectional data during pregnancy of 21,785 most recent births within five years preceding the 2018 Nigeria Demographic Health Survey was used. The number of ANC contacts was categorised into “None”, “1–3”, “4–7” and “8 or more” contacts based on subsequent WHO guidelines. Descriptive statistics, bivariable and multivariable multinomial logistic regression was used at *p* = 0.05.

**Results:**

About 25 % of the women had no ANC contact, 58 % had at least 4 contacts while only 20 % had 8 or more ANC contacts. The highest rate of 8 or more ANC contacts was in Osun (80.2 %), Lagos (76.8 %), and Imo (72.0 %) while the lowest rates were in Kebbi (0.2 %), Zamfara (1.1 %) and Yobe (1.3 %). Respondents with higher education were twelve times (adjusted relative risk (aRR): 12.46, 95 % CI: 7.33–21.2), having secondary education was thrice (aRR: 2.91, 95 % CI: 2.35–3.60), and having primary education was twice (aRR: 2.17, 95 % CI: 1.77–2.66) more likely to make at least 8 contacts than those with no education. Respondents from households in the richest and middle wealth categories were 129 and 67 % more likely to make 8 or more ANC contacts compared to those from households in the lowest wealth category respectively. The likelihood of making 8 ANC contacts was 89 and 47 % higher among respondents from communities in the least and middle disadvantaged groups, respectively,  compared to the most disadvantaged group. Other significant variables were spouse education, health care decision making, media access, ethnicity, religion, and other community factors.

**Conclusions:**

Compliance with WHO guidelines on the minimum number of ANC contacts in Nigeria is poor. Thus, Nigeria has a long walk to attaining sustainable development goal’s targets on child and maternal health. We recommend that the maternal and child health programmers should review existing policies and develop new policies to adopt, implement and tackle the challenges of adherence to the WHO recommended minimum of 8 ANC contacts. Women's education, socioeconomic status and adequate mobilization of families should be prioritized. There is a need for urgent intervention to narrow the identified inequalities and substantial disparities in the characteristics of pregnant women across the regions and states.

## Introduction

Nigeria, the largest and the most populous country in sub-Saharan Africa (SSA), has unimpressive maternal and child health indicators. These indicators ranked among the worst in the world despite years of intervention efforts by the government and other stakeholders [1–3]. According to the WHO and a group of other international agencies, one in every four (23 %) global maternal deaths in 2017 occurred in Nigeria. This indicated that a pregnant woman in Nigeria had a one in 21 chance of dying from pregnancy and childbirth-related causes, compared to a one in 38 in SSA, 1:190 globally, 1:3,100 in North America and 1:11,700 in Western Europe [4, 5]. A 2017 maternal health situation analysis indicated that for 17 years in Nigeria, only about 24 % was shed off from the maternal mortality ratio (1200 in 2000 and 917 in 2017) [5].

While pregnancy-related maternal mortality (PRMM) has remained relatively the same over the past 18 years, there has been a clear decrease in childhood mortality in Nigeria over the same period [1, 6]. The PRMM rose from 545 (95 % Confidence Interval (CI): 484–629) per 100,000 live births in 2001–2008, to 576 (95 % CI: 500–653) in 2006–2013, and reduced to 556 in 2011–2018. The confidence intervals of these estimates overlapped, an indication of non-significance [7, 8]. According to these recent statistics, under-5 mortality and infant mortality rates have reduced from 157 deaths per 1,000 live births in 2008 to 132 deaths per 1,000 live births in 2018, and from 75 deaths per 1,000 live births to 67 deaths per 1,000 live births over the same period, respectively [7, 8]. Despite recent improvements in childhood mortality rates in Nigeria, the country remained one of the countries with the worst health indicators [5].

Whereas, having early contacts with antenatal care (ANC) providers, and more importantly, having sufficient numbers of ANC contacts could enable early detection of any negative condition and thereby enhance pregnancy outcomes and improve child survival, ANC uptake in Nigeria has remained low [9–12]. ANC contacts also enable adequate health education and promotion on signs of danger during pregnancy, prevention-of-mother-to-child-transmission of HIV/AIDS, reduce chances of low birth weight, the risk of preterm delivery, and perinatal mortality [2, 13, 14]. ANC is effective in reducing morbidity and/or mortality among both expectant mothers and babies before, during or after delivery [12, 15–17].

The WHO is committed to global intervention actions to ensure improved maternal and child healthcare through ANC utilization [12, 18]. WHO recommended early ANC attendance for pregnant women and set the minimum number of ANC contacts as well as the minimum standards for the effective operationalization of the healthcare components to be offered to expectant women [18]. In 2016, reviews were made to these standards to ensure further strengthening of the ANC program as follow-up strategies to the realization of the relevant goal of the sustainable development goals (SDG) by 2030. The number of ANC contacts required to guarantee a safe delivery was increased from a minimum of 4 to 8 contacts in 2016, the first ANC contact was to be during the first trimester of pregnancy, and emphasis was laid on compulsory administration of specific components of ANC such as blood and urine tests, use of intermittent preventive treatment (IPTp) by women during pregnancy etc. [12]. Following the WHO guideline, the Nigerian government published an orientation package on its 2017 ANC model, and consequently adopted the guidelines with directives to ANC providers to operationalize it [19].

A study on barriers to ANC attendance in Nigeria identified poverty, rural residence, currently married status of women, and a low level of education among women as associated factors [13]. The study found that poor and rural-resident women were more likely to experience barriers to ANC access than their counterparts who were richer and resident in urban centres [13, 20–22]. Also, Fagbamigbe et al. affirmed the role of educational attainment in ANC utilization in Nigeria [13]. Several studies have affirmed the link between poor maternal and infant health outcomes and inadequacy or outright lack of antenatal care [2, 23–26]. Also, other studies have connected the geographical region of women’s residence with individual and community-level factors associated with the number of ANC contacts [22, 23, 27–29]. Women's age has been associated with ANC contacts made, as fewer ANC contacts were found among pregnant teenagers [21, 27, 30, 31]. Spousal level of education and preceding birth intervals have also been reported as predictors of the number of ANC contacts made [22, 32–34].

Studies in the past have shown that low ANC coverage in Nigeria and other developing countries may be due to inadequate provision of healthcare facilities and congestion in the available ones [13, 35–38]. However, there have been improvements in the primary healthcare system in recent times through the Nigerian State Health Investment Programme (NSHIP) – a World Bank sponsored project [39]. The improvements include the establishment of stronger management systems and supervision processes, an increase in staff knowledge, motivation and engagement, and the overall improvement in working conditions and higher uptake of services at NSHIP facilities [39, 40]. The major objectives of NSHIP are to improve service delivery, through high impact maternal and child health interventions and care, at selected facilities in the participating states. Performance-based financing (PBF) and decentralized facility financing (DFF) were also instituted. With the pilot completed in 2011, the project ran from 2013 to 2018 [40]. With the NSHIP interventions, one would have expected improvement in child and maternal outcomes especially in the rural areas, where low ANC attendance is more associated with ineffective service delivery than in the urban centres [23, 27, 41].

The new ANC framework, tagged “Antenatal Care for Positive Pregnancy Experience” by WHO in 2016, combined with the improved healthcare system by the Nigerian government was expected to have yielded substantial better maternal, infant and child health outcomes [12, 19]. The expected improvements did not happen and it is worth examining why. It is therefore imperative to assess the level of compliance with the recommended standards of ANC in Nigeria. The assessment could inform policy for implementation of the WHO ANC guidelines in the country. This study was motivated by the need to monitor and evaluate the progress made in Nigeria to attain the SDG-3 that was set to “ensure healthy lives and promote well-being for all at all ages” [42]. In particular, we aimed to determine how close Nigeria is towards meeting the SDG-3 target 3.1: “By 2030, end preventable deaths of new-borns and children under 5 years of age, with all countries aiming to reduce neonatal mortality to at least as low as 12 per 1,000 live births and under-5 mortality to at least as low as 25 per 1,000 live births” [42]. Therefore, the objective of this study is to examine compliance with the WHO recommended standards on minimum ANC contacts during pregnancy in Nigeria. In this study, we assessed the distribution of the number of ANC contacts and identified the associated factors in Nigeria using nationally representative data. We answered one of the SDG’s calls for continuous monitoring and evaluation of countries’ closeness to the achievement of the SDGs. While most studies on ANC contacts in Nigeria were hitherto restricted to national and regional levels, the current study explored the distribution of ANC contacts across each state. National and regional analysis conceals the state-level scenarios. The regions in Nigeria are not administrative units but the states are. The State governments have decision-making power and are better-positioned to use state-specific findings. The study suggested pathways to increase the number of ANC contacts as a strategy to improve maternal, child and infant health outcomes in Nigeria. This study bridged the assessment of compliance with the old (2002) and new (2016) WHO ANC guidelines.

## Methods

### Study setting

**Fig. 1:**
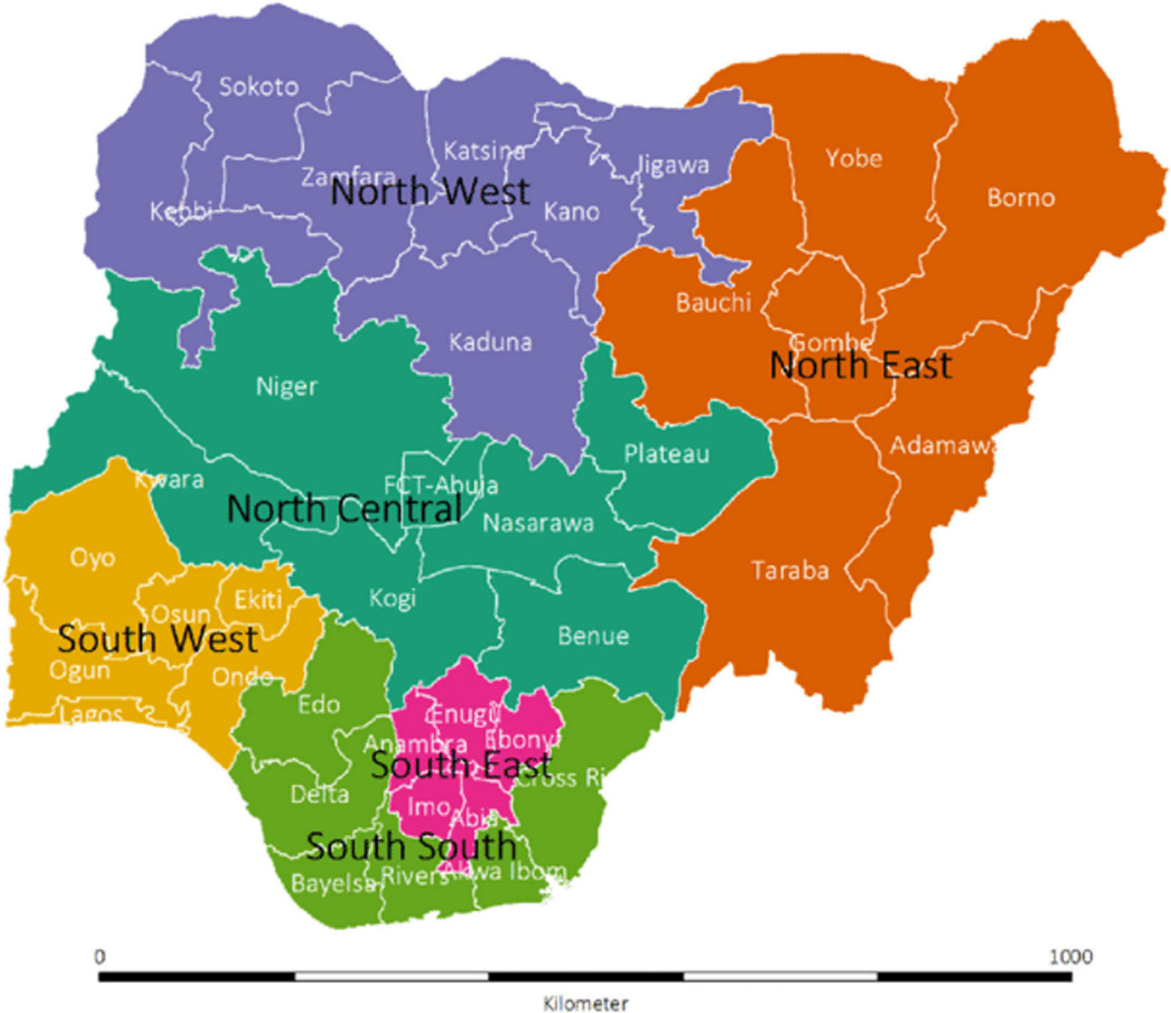
Map of Nigeria showing the 36 states and the federal capital territory (FCT), by the geopolitical zones

Nigeria has 36 states and the Federal Capital Territory (FCT). The states are further grouped into 6 regions as shown in Fig. 1. Although the regions have no administrative functions, people within each region are deemed to have similar characteristics, culture, ethnicity, vegetation etc. The States are further subdivided into local government areas (LGAs). The LGAs are subdivided into wards which are political/health units. The wards are also known as enumeration areas (EAs). Following the new 2016 WHO ANC guideline, the Federal Ministry of Health in Nigeria developed an orientation package for a new ANC model in Nigeria in 2017. The model marked the transition from a minimum of 4 visits to a minimum of 8 contacts [19]. With an emphasis on contacts rather than visits, ANC providers were trained, orientated and directed to operationalize it [19].

## Data source

We used cross-sectional and nationally representative secondary data from the 2018 Nigeria Demographic Health Survey (NDHS) conducted by ICF International Calverton, Maryland, USA in conjunction with the Nigeria National Population Commission (NPC) [8]. DHSs are conducted periodically among consenting Low and Middle-Income Countries (LMIC). The surveys provide information about population, demographics, sexuality and other reproductive health indicators among men and women of reproductive age.

## Sampling techniques

The 2018 NDHS used the 2006 Population and Housing Census of the Federal Republic of Nigeria as the sampling frame. The primary sampling unit (PSU) in the survey are the EAs. In each state, samples of EAs were selected independently through a two-stage selection procedure. Firstly, about 38 EAs were selected with probability proportional to the size of EAs within each state. Secondly, a full listing of households in all selected EAs from the first stage was carried out to serve as the sampling frame for the selection of households. A fixed number of 30 households were selected from the sampling frame of each EA by an equal probability systematic sampling. All eligible women of reproductive age (15–49 years) in each of the selected households were interviewed. Due to the non-proportional allocation of the sample to the different states, LGAs and EAs, as well as the possible differences in response rates in each EA, sampling weights were applied to all our analysis. A total of 41,821 women aged 15–49 years were interviewed [8].

## The data

All respondents (women aged 15–49 years) were asked if they had any pregnancy or birth within 5 years preceding the survey. Those who had had at least one birth were asked questions relating to conception, pregnancy-related care including the number of ANC contacts made, the time ANC began, ANC provider used etc. for the pregnancy starting from the most recent. Our analysis was based on the information provided by the respondents on their most recent births. A total of 21,785 women provided relevant history and antenatal care information about their most recent births.

## Variables

### Outcome variable

The number of ANC contacts made by women during the pregnancy of their most recent births within five years preceding the survey is the outcome variable. We divided the number of ANC contacts into four categories: No contact, 1–3 contacts, 4–7 contacts and 8 or more contacts. The categorization was based on the earlier recommendation of a minimum of 4 ANC visits in 2006 and the later recommendation of a minimum of 8 ANC contacts in 2016 with skilled ANC providers [12]. The skilled ANC providers included doctors, nurses/midwives, and auxiliary nurses/midwives [8] according to the new WHO guideline. Wherein, “No contact” meant no contact at all, “1–3” implies some contacts but do not meet the old guideline, “4–7” met the old guideline but not the new one while 8 + are those that meet the new guideline.

To explore the level of no ANC contacts across the states in Nigeria, we used the 15 % global “no antenatal contact” prevalence to group the states into two: “below 15 %” and “>=15 %” [43, 44]. For the visualization of the state performance regarding the proportion of women who had 8 + contacts during the pregnancy of their most recent births, the States were grouped into having 0–25 %, 26–50 %, 51–75 % and 76–100 % 8 + ANC contacts.

## Independent variables

Based on existing literature [15, 18, 23, 31, 35, 45], and Anderson’s model for health care utilization [46], the independent variables are maternal age (15–19,20–24, 25–29, 30–39, 40–49 years), education (no education, primary, secondary and higher), spouse education (no education, primary, secondary and higher), employment status (currently employed or not), spouse employment status (currently employed or not), access to media (at least one of radio, television, or newspaper), household wealth index (low, middle, and high), women’s autonomy using who decides respondents health care access as a proxy (respondent alone, respondent/spouse, and spouse alone). Others are birth interval (firstborn, < 36 months, and > = 36 months), birth order (1, 2, 3, 4 and 5+), children ever born (1–2, 3–4, 4+), current marital status (currently married or living together (living in union), divorced/separated/widowed, never married), place of residence (rural/urban), religion (Islam, Christianity, others), and ethnicity (Hausa/Fulani, Igbo, Ibibio, Yoruba and others). Others are family mobility (had stayed less than five years at residence or not), wanted child when became pregnant (then, later or not more), household headship (male or female), health insurance coverage (yes or no), acceptance of wife-beating (yes or no), region (North Central, North East, North West, South East, South-South and South West).

We generated four community-level factors to identify community characteristics that could affect the numbers of ANC contacts made during pregnancy. The word “community” was used to connote the stratum (EAs or geographical clustering) which are the PSU within the DHS sampling frame. The four factors are the community poverty rate (high or low), community unemployment rate (high or low), community illiteracy rate (high or low), and community media access rate (high or low) as the proportion of respondents within each community who are poor, unemployed, illiterates, and with no media access respectively. We computed the neighbourhood socioeconomic (SES) disadvantage composite score from community poverty rate, community unemployment rate, community illiteracy rate, and community media access rate using principal component analysis. The neighbourhood SES disadvantage composite score was grouped into lowest, middle and highest.

## Statistical methods

We analysed the data using descriptive statistics, chi-square bivariable test of relationships and multivariable multinomial logistic regression in Stata version 16 (Stata Corp, Texas, USA). Frequency tables showing percentages were used to describe the distribution of study respondents’ characteristics and we cross-classified the outcome variable by the respondents’ characteristics (Table 1). Charts and maps were used to visualize our findings.

We used the multinomial logistic command in Stata to carry out a multivariable regression analysis of the factors associated with the outcome variables. At the bivariable level, we identified all variables that were significant at *p* < 0.20 which were then included in the multivariable regression from where we identified the adjusted risk ratios (aRR) of characteristics associated with the outcome variables. The farther the aRR is from one, the higher the risk.

The multinomial logistic regression model finds the maximum-likelihood estimates of the probability of success. Let *X*_*j*_ be the vector of independent variables, augmented by 1, and *b* as the corresponding estimated parameter vector. The odds ratio, estimated as the ratio of odds of success divided by the odds of failure, is the *i*^*th*^ coefficient is *ϕ*_*i*_ = exp(b_i_) with standard error ${ s}_{i}^{\varphi }={\varphi }_{i}{s}_{i}$, where ${s}_{i}$is the standard error of ${b}_{i}$ estimated by logit. Assuming that the predicted index of the *j*^*th*^ observation is defined as ${X}_{i}b$, then the predicted probability of a positive outcome is

$${P}_{j}\left({y}_{j}\ne 0 \right| {X}_{j})=\frac{\text{e}\text{x}\text{p}\left({X}_{i}b\right)}{1+\text{e}\text{x}\text{p}\left({X}_{i}b\right)}$$

The likelihood function for the logit is

$$lnL=\sum _{j{\epsilon}S}{w}_{j} lnF\left({x}_{j}b\right)+ \sum _{j\ni S}{w}_{j} ln\left\{1-F\left({x}_{j}b\right)\right\}$$

Where S is the set of all observations j, such that ${y}_{j}\ne 0, F\left(z\right)={e}^{z}$/(1+${e}^{z}$), and ${w}_{j}$ are the operational weights

Multinomials are used for the categorical dependent variable which must have a minimum of three categories. Let us assume that y has three outcomes 1, 2, 3 and 4 whereby “4” is not necessarily greater than “1” or “2” or “3”. Then multinomial logistic regression was used to model nominal outcome variables, in which the log odds of the outcomes are modelled as a linear combination of the predictor variables [47]. It fits maximum likelihood models with discrete dependent variables with more than two outcome categories and these categories do not necessarily have a natural ordering.

Considering the categories 1, 2, 3, 4, ……., m recorded in y, and the explanatory variables X. Assuming that y = 1 if a respondent has no ANC attendance, y = 2 if a respondent made 1 to 3 ANC contacts, y = 3 if a respondent made 4 to 7 ANC contacts and y = 4 if a respondent made 8 or more ANC contacts. A set of coefficients, β^(1)^, β^(2)^, β^(3)^ and β^(4)^ corresponding to each category is estimated:

1 $$P(y=1)=\frac{{e}^{X{\beta }^{\left(1\right)}}}{{e}^{X{\beta }^{\left(1\right)}}+ {e}^{X{\beta }^{\left(2\right)}}+{ e}^{X{\beta }^{\left(3\right)}}+{e}^{X{\beta }^{\left(4\right)}}}$$

2 $$P(y=2)=\frac{{e}^{X{\beta }^{\left(2\right)}}}{{e}^{X{\beta }^{\left(1\right)}}+ {e}^{X{\beta }^{\left(2\right)}}+ {{e}^{X{\beta }^{\left(3\right)}}+ e}^{X{\beta }^{\left(4\right)}}}$$

3 $$P(y=3)=\frac{{e}^{X{\beta }^{\left(3\right)}}}{{e}^{X{\beta }^{\left(1\right)}}+ {e}^{X{\beta }^{\left(2\right)}}+ {e}^{X{\beta }^{\left(3\right)}}+ {e}^{X{\beta }^{\left(4\right)}}}$$

4 $$P(y=4)=\frac{{e}^{X{\beta }^{\left(4\right)}}}{{e}^{X{\beta }^{\left(1\right)}}+ {e}^{X{\beta }^{\left(2\right)}}+ {e}^{X{\beta }^{\left(3\right)}}+ {e}^{X{\beta }^{\left(4\right)}}}$$

The model is unidentified since there are more than one solutions for β^(1)^, β^(2)^, β^(3)^ and β^(4)^ that can result in the same probabilities for y = 1, y = 2 and y = 3. One of the coefficients β^(1)^, β^(2)^, β^(3)^ and β^(4)^ would then be set arbitrarily to 0. If we set β^(1)^ = 0, the remaining coefficients β^(2)^, β^(3)^ and β^(4)^ will measure the change relative to the y = 1 group. The coefficients will differ because they have different interpretations, but the predicted probabilities for y = 1, 2, 3 and 4 will still be the same.

Setting ${\beta }^{\left(1\right)}=0$, the set of Eqs. (1), (2), (3) and (4) equation become

5 $$P(y=1)=\frac{1}{1+{e}^{X{\beta }^{\left(2\right)}}+{e}^{X{\beta }^{\left(3\right)}}+{e}^{X{\beta }^{\left(4\right)}}}$$

6 $$P(y=2)=\frac{1}{{e}^{X{\beta }^{\left(1\right)}}+1+{e}^{X{\beta }^{\left(3\right)}}+{e}^{X{\beta }^{\left(4\right)}}}$$

7 $$P(y=3)=\frac{{e}^{X{\beta }^{\left(3\right)}}}{{e}^{X{\beta }^{\left(1\right)}}+{e}^{X{\beta }^{\left(2\right)}}+1+{e}^{X{\beta }^{\left(4\right)}}}$$

8 $$P(y=4)=\frac{{e}^{X{\beta }^{\left(3\right)}}}{{e}^{X{\beta }^{\left(1\right)}}+{e}^{X{\beta }^{\left(2\right)}}+{e}^{X{\beta }^{\left(3\right)}}+1}$$

respectively.

The relative probability of y = 2 to the base outcome is

9 $$\frac{P(y=2)}{P(y=1)}={e}^{X{\beta }^{\left(2\right)}}$$

Which is the relative risk ratio. Assuming that *X* and ${\beta }_{k}^{\left(2\right)}$are vectors equal to (${x}_{1}+{x}_{2}+\dots {x}_{i}\dots .+{x}_{k})$ and $({\beta }_{1}^{\left(2\right)}+{\beta }_{2}^{\left(2\right)}+\dots {+\beta }_{i}^{\left(2\right)}+\dots {\beta }_{k}^{\left(2\right)})$. Then, the ratio of the relative risk for a one-unit change in x_t_ is

10 $$\frac{{e}^{{\beta }_{1}^{\left(2\right)}{x}_{1}}+\dots .+{e}^{{\beta }_{i}^{\left(2\right)}{x}_{(i+1)}}+\dots \dots +{e}^{{\beta }_{k}^{\left(2\right)}{x}_{k}}}{{e}^{{\beta }_{1}^{\left(2\right)}{x}_{1}}+\dots .+{e}^{{\beta }_{i}^{\left(2\right)}{x}_{i}}+\dots \dots +{e}^{{\beta }_{k}^{\left(2\right)}{x}_{k}}}={e}^{X{\beta }^{\left(2\right)}}$$

Thus, the exponentiated value of a coefficient is the relative-risk ratio (RRR) for a one-unit change in the corresponding variable (risk is measured as the risk of the outcome relative to the base outcome). This is the ratio of the probability of choosing one outcome category over the probability of choosing the baseline category and it is also sometimes referred to as odds [47, 48]. For the multivariable model, we checked for multicollinearity among the independent variables using the “collin” command in Stata 16 and removed those that were colinear. We included only the variables with a variance inflation fraction lower than 2.3 in the analysis.

## Results

In all, 21,785 women provided information about their most recent births during the 5 years preceding the survey.

## Numbers of ANC contacts

**Fig. 2:**
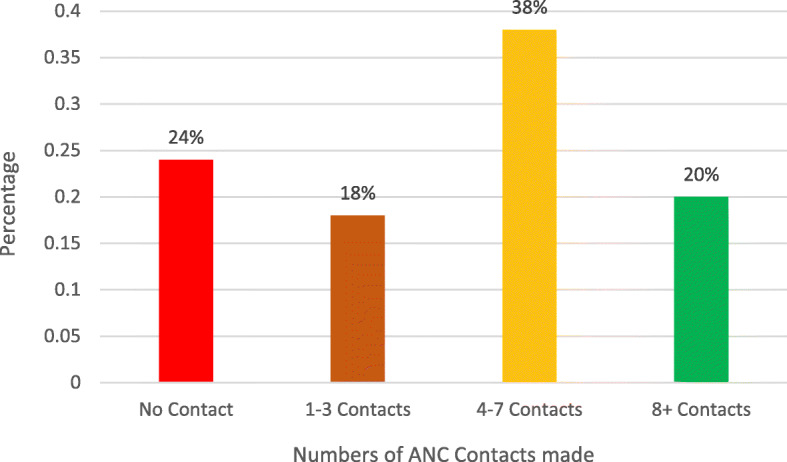
Distribution of the number of antenatal care contacts during the most recent pregnancy

About 25 % of the women had no contact with any ANC provider, 37.5 % had 4 to 7 visits and 20 % met the new WHO-recommended minimum of 8 ANC contacts (Table 1; Fig. 2). In all, 58 % met the old WHO recommendation of a minimum of 4 contacts while only 18 % made 1–3 contacts. About 10 % of mothers aged 15–19 made 8 or more ANC contacts, 14.4 % of mothers aged 20–24 made 8 or more ANC contacts, women with no education (4.2 %), where spouse alone decided health care access (11 %), women with no media exposure (6 %), Hausa/Fulani women (4 %), women from low wealth households (5 %), unemployed women (11 %) North Eastern women (4 %), North-Western women (4 %) and rural women (11 %) as shown in Table 1. The proportion of pregnant women who made 1–3 and 4–7 contacts were generally higher among pregnant women from the communities with low rates of poverty than those from communities with high rates (1–3 contacts: 22.4 % v 14.5 %, 4–7 contacts: 48.2 % vs. 31.2 %) but having at least 8 contacts was lower in the communities with lower community poverty (10.7 % vs. 25.9 %). Similar differences were noticeable among the pregnant women from the communities with low rates of unemployment, illiteracy and media barrier than those from communities with high rates. About 46 % of the pregnant women from the least disadvantaged communities had at least 8 ANC contacts compared with 12 % among those from communities with a mid-level disadvantage and less than 3 % among those from most disadvantaged communities. All the explanatory variables considered were significantly associated with the number of ANC contacts made (*p* < 0.001).

**Table 1:** Association between respondents’ characteristics and number of ANC contacts during the pregnancy of their most recent birth

Characteristics		Number of Antenatal Contact (%)				χ^2^	*p*-value
	**N(%)**	**None**	**1–3**	**4–7**	**8+**		
**Age of mothers (years)**						387.7*	0.000
15–19	1203(5.5)	32.9	23.2	34.4	9.5		
20–24	4186(19.2)	26.5	19.3	39.8	14.4		
25–29	5647(25.9)	23.3	18.3	38.2	20.2		
30–39	8345(38.3)	22.3	15.9	36.8	25.1		
40–49	2404(11.0)	29.6	14.8	36.4	19.2		
**Highest educational level**						6052.3*	0.000
No education	9683(44.5)	43.4	22.0	30.4	4.2		
Primary	3272(15.0)	16.6	19.2	44.7	19.5		
Secondary	6921(31.8)	8.5	12.9	44.0	34.6		
Higher	1908(8.8)	1.0	6.8	39.0	53.2		
**Spouse’s highest education**						4813.3*	0.000
No education	7313(36.1)	47.6	21.4	26.6	4.3		
Primary	2822(13.9)	19.3	20.1	42.8	17.8		
Secondary	6975(34.5)	11.9	14.4	43.5	30.2		
Higher	3126(15.4)	4.1	13.5	44.1	38.2		
**Who decides respondent’s healthcare**						2290.2*	0.000
Respondent alone	1996(9.8)	13.5	14.2	32.5	39.7		
Respondent and Spouse	6324(31.1)	12.5	13.1	42.9	31.5		
Spouse alone	12,039(59.1)	33.2	20.5	35.3	11.0		
**Media exposure**						3115.5*	0.000
Unexposed to media	8196(37.6)	41.5	21.6	31.0	5.9		
Exposed to media	13,589(62.4)	14.5	14.9	41.6	29.1		
**Ethnicity**						5900.0*	0.000
Hausa/Fulani	9572(43.9)	37.3	21.8	36.5	4.4		
Yoruba	2743(12.6)	4.7	4.5	29.1	61.6		
Igbo	2666(12.2)	3.8	9.9	42.4	44.0		
Ibibio	381(1.8)	17.7	15.4	45.0	21.9		
Others	6422(29.5)	23.1	19.5	40.3	17.0		
**Religion**						3027.9*	0.000
Islam	13,373(61.4)	32.7	21.0	36.2	10.2		
Christians	8295(38.1)	11.6	11.6	39.4	37.1		
Others	117(0.5)	34.5	24.7	35.7	5.1		
**Marital Status**						44.8*	0.000
Never married	511(2.3)	21.8	15.7	33.6	28.9		
Living in a union	20,520(94.2)	25.0	17.6	37.4	20.0		
Widowed/Divorced/Separated	754(3.5)	20.3	14.1	44.6	21.0		
**Wealth status**						5729.2*	0.000
Low	6912(31.7)	45.3	21.3	28.9	4.7		
Middle	7036(32.3)	24.1	20.4	41.5	14.0		
Richest	7837(36.0)	6.9	11.2	41.8	40.2		
**Children ever-born**						717.3*	0.000
1 or 2 births	7637(35.1)	19.7	15.2	38.5	26.6		
3 or 4 births	6133(28.2)	22.1	17.2	37.8	22.9		
More than 4 births	8015(36.8)	31.5	19.8	36.4	12.2		
**Birth order**						753.9*	0.000
First	3735(17.1)	18.6	15.8	39.4	26.2		
Second	3902(17.9)	20.8	14.6	37.6	27.1		
Third	3339(15.3)	20.7	16.3	37.7	25.3		
Fourth	2794(12.8)	23.6	18.3	38.0	20.0		
Fifth or higher	8015(36.8)	31.5	19.8	36.4	12.2		
**Birth interval**						133.7*	0.000
First Birth	3735(17.1)	18.6	15.8	39.4	26.2		
< 36 months	10,453(48.1)	26.9	17.8	37.7	17.5		
>=36 months	7558(34.8)	24.5	16.6	37.4	21.5		
**Current employment status**						876.6*	0.000
Employed	14,898(68.4)	20.1	16.4	38.9	24.6		
Unemployed	6887(31.6)	34.7	19.7	34.5	11.0		
**Spouse current employment statusment**						954.1*	0.000
Employed	15,712(72.1)	20.2	16.5	39.1	24.2		
Unemployed	6073(27.9)	36.5	19.8	33.5	10.1		
**Household head sex**						177.3*	0.000
Male	19,659(90.2)	25.6	17.8	37.3	19.3		
Female	2126(9.8)	16.7	14.0	39.9	29.5		
**Wanted Last child**						141.6*	0.000
Then	19,142(87.9)	25.6	17.6	37.6	19.2		
Later	1919(8.8)	17.4	17.0	37.6	28.0		
Never	724(3.3)	22.8	13.5	35.8	27.9		
**Family Mobility**						452.8*	0.000
5 + yr	18,201(83.5)	26.6	18.1	37.3	18.0		
Less stable 0-4yr	3584(16.5)	15.4	14.0	38.8	31.8		
**Have health Insurance**						189.3	0.000
No	21,306(97.8)	25.1	17.6	37.6	19.7		
Yes	479(2.2)	9.2	10.8	36.3	43.7		
**Wife Beating acceptable**						1457.9*	0.000
No	14,897(68.4)	19.0	16.0	38.9	26.1		
Yes	6887(31.6)	37.0	20.6	34.6	7.9		
**Community Poverty**						1327.7*	0.000
Low	8018(36.8)	18.8	22.4	48.2	10.7		
High	13,767(63.2)	28.3	14.5	31.2	25.9		
**Community Illiteracy**						1181.8*	0.000
Low	8106(37.2)	16.0	21.4	48.6	14.0		
High	13,679(62.8)	30.0	15.1	30.9	24.0		
**Community Unemployment**						129.6*	0.001
Low	9348(42.9)	23.2	19.4	39.9	17.6		
High	12,437(57.1)	26.0	16.0	35.8	22.2		
**Community Media barrier**						817.1*	0.000
Low	9508(42.9)	17.3	19.9	45.7	17.1		
High	12,277(57.1)	30.6	15.5	31.1	22.7		
**Community SES Disadvantage**						7220.1*	0.000
Lowest	7542(34.6)	6.7	8.3	38.9	46.0		
Middle	7190(33.0)	19.7	22.8	46.0	11.6		
Highest	7053(32.4)	48.5	21.4	27.6	2.6		
**Region**							
North central	3014(13.8)	27.8	17.6	40.3	14.3	6300.1*	0.000
North East	3841(17.6)	28.5	27.5	40.3	3.7		
North West	7602(34.9)	36.3	21.4	38.1	4.2		
South East	2126(9.7)	3.8	11.9	45.8	38.5		
South South	2007(9.2)	18.8	8.6	33.2	39.3		
South West	3194(14.7)	5.9	4.2	26.7	63.2		
**Residence**							
Urban	8659(39.8)	10.4	13.6	40.6	35.5	2200.1*	0.000
Rural	12,126(60.2)	34.0	20.0	35.6	10.5		
Total	21,785	24.5	17.5	37.6	20.4		

*significant χ^2^at 0.05

The performance of the states in the number of ANC contact is presented in Table 2. The highest rates of 8 or more ANC contacts were in Osun (80.2 %), Lagos (76.8 %), and Imo (72.0 %) while the lowest rates were in Kebbi (0.2 %), Zamfara (1.1 %) and Yobe (1.3 %) as shown in Table 2.

**Table 2:** Distribution of the number of antenatal care contacts during the pregnancy of their most recent birth by states in Nigeria

State*	n	No contact	1–3 contacts	4–7 contacts	8 + contacts
Osun	407	1.2	3.2	15.3	80.2
Lagos	1,129	4.6	2.0	16.5	76.8
Imo	403	2.5	10.9	14.7	72.0
Ondo	310	4.6	6.7	23.2	65.5
Edo	269	10.0	5.0	20.4	64.5
Ekiti	225	6.9	6.5	25.9	60.7
Rivers	603	12.4	5.8	26.3	55.4
Oyo	702	12.1	5.5	34.1	48.2
Enugu	315	3.8	8.0	45.1	43.2
Kwara	358	25.4	8.6	24.4	41.7
Abia	257	3.8	5.7	48.8	41.7
Delta	405	23.3	3.6	33.3	39.9
Ogun	421	3.8	5.0	52.5	38.7
Anambra	661	3.4	10.9	59.2	26.5
Kogi	297	17.8	9.5	47.5	25.3
Akwa Ibom	358	19.3	14.4	44.0	22.3
Ebonyi	490	5.6	19.8	52.6	22.0
FCT, Abuja	147	12.3	13.4	58.2	16.1
Adamawa	515	15.5	17.7	52.1	14.7
Cross River	229	16.0	18.0	51.6	14.3
Benue	633	25.4	18.3	42.1	14.1
Nasarawa	327	22.4	10.4	54.1	13.1
Plateau	413	24.1	20.1	47.2	8.6
Kaduna	1445	30.0	15.9	46.1	8.0
Kano	1672	16.4	32.5	44.2	6.9
Bayelsa	143	57.7	10.7	25.4	6.2
Sokoto	605	53.1	16.2	27.0	3.7
Taraba	492	20.4	29.8	46.5	3.4
Jigawa	893	20.5	31.6	45.0	2.9
Bauchi	914	33.1	24.6	39.9	2.4
Katsina	1432	47.0	14.4	36.6	2.0
Niger	839	40.8	26.1	31.5	1.7
Gombe	442	25.5	30.0	42.9	1.6
Borno	728	37.6	28.0	33.0	1.4
Yobe	751	30.2	34.0	34.4	1.3
Zamfara	843	63.7	10.3	24.9	1.1
Kebbi	712	47.9	24.7	27.2	0.2
Total	21,785	24.8	17.4	37.5	20.3

*ordered by 8 + contacts

**Fig. 3:**
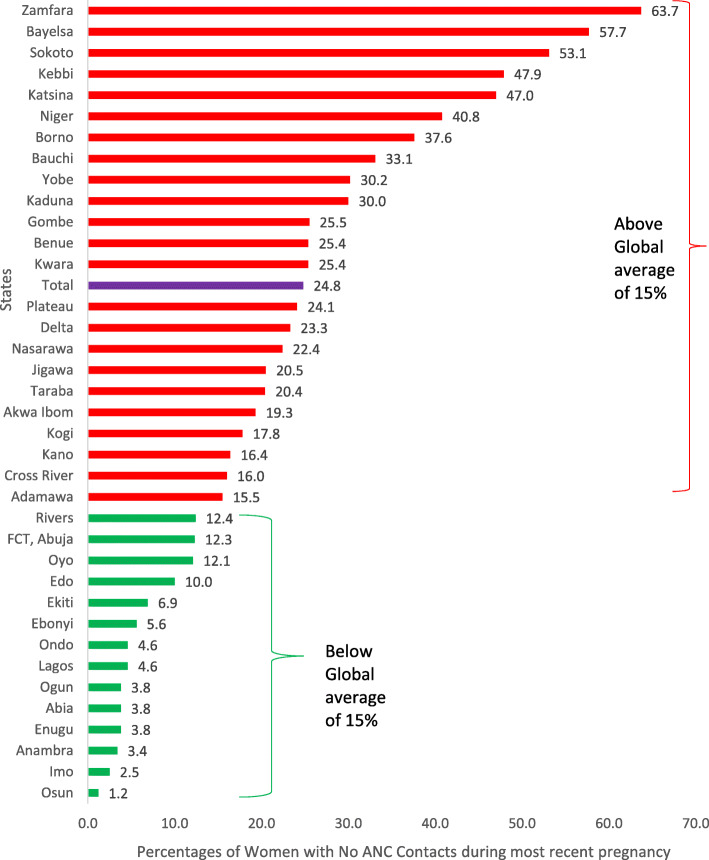
Distribution of women with no ANC contacts during most recent births by States

In comparison with the 15 % global prevalence of pregnancies with no ANC contact, only 14 of the 37 states of Nigeria had less than 15 % of pregnant women made no ANC contact as shown in Fig. 3.

**Fig. 4:**
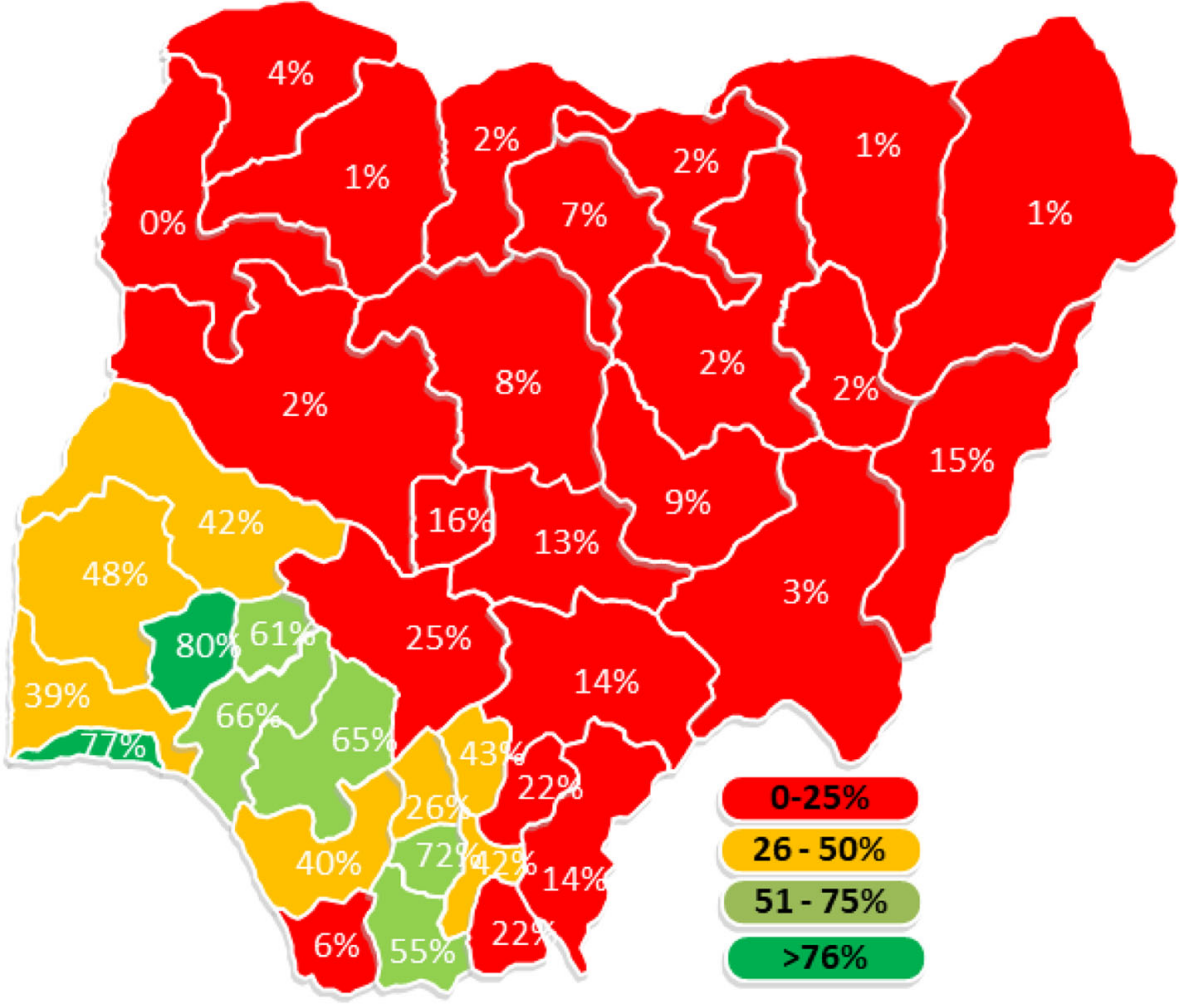
Distribution of percentages that had 8 + ANC contacts during pregnancy for the most recent birth by States in Nigeria (Authors drawing)

In all, the proportion of women that made the recommended minimum of 8 ANC contacts was ≤ 25 % in most (23 out of 37) of the States in Nigeria, while only Osun and Lagos state had more than 75 % as shown in Fig. 4.

## Determinants of the number of ANC contacts made

During pregnancy, mothers aged 40–49 years (adjusted Risk Ratio (aRR): 1.62, 95 % CI: 1.10–2.38), those aged 30–39 years (aRR: 1.83, 95 % CI: 1.29–2.60) and those aged 25–29 years (aRR: 1.40, 95 % CI: 1.01–1.95) were 62 %, 83 and 40 % respectively more likely to make 8 or more ANC contacts relative to those aged 15–19 years as shown in Table 3. Respondents with higher education were 12 times (aRR: 12.46, 95 % CI: 7.33–21.2), those with secondary education were thrice (aRR: 2.91, 95 % CI: 2.35–3.60), and those with primary education were twice (aRR: 2.17, 95 % CI: 1.77–2.66) more likely to make 8 or more ANC contacts relative than those with no education. Mothers in North East (28 %), South East (223 %) and South West (284 %) more likely to make 8 or more ANC contacts relative to those in the North West. Also, respondents from households in the richest and middle wealth categories were 129 and 67 % more likely to make 8 or more ANC contacts compared to those from households in the low wealth category respectively. The likelihood of making 8 ANC contacts was 89 and 47 % higher among respondents from communities in the least and middle disadvantaged group. Pregnant women from the least disadvantaged communities were 50 % (aRR:1.50, 95 % CI: 1.17–1.93), 89 % (aRR:1.89, 95 % CI: 1.52–2.36), and 89 % (aRR: 1.89, 95 % CI: 1.38–2.57) to make 1–3, 4–7 and at least 8 ANC contacts respectively than those from most disadvantaged communities. Other significant variables to making 8 or more ANC contacts were spouse education, the person who decides the respondent’s health care, media access, ethnicity, religion, children ever born, birth interval, spouse current employment status, and place of residence. All the variables in Table 3 were significant at *p* < 0.20 in the bivariable logistic regression and chi-square analysis presented in Table 1.

**Table 3:** Adjusted risk ratios for having recommended number of ANC contacts (NDHS 2018)

Characteristics	1 to 3 contacts		4 to7		8 and more contacts	
	aRR(95 % CI)	sig	aRR(95 % CI)	sig	aRR(95 % CI)	sig
**Age of mothers (years)**						
15–19	Reference					
20–24	0.96 (0.77–1.20)	0.73	1.11 (0.91–1.36)	0.30	1.19 (0.86–1.65)	0.28
25–29	0.99 (0.78–1.26)	0.92	1.24 (0.99–1.54)	0.06	1.40 (1.01–1.95)	0.04
30–39	0.88 (0.68–1.14)	0.34	1.29 (1.02–1.64)	0.04	1.83 (1.29–2.60)	0.00
40–49	0.61 (0.46–0.82)	0.00	1.10 (0.84–1.43)	0.48	1.62 (1.10–2.38)	0.01
**Highest educational level**						
No education	Reference					
Primary	1.69 (1.45–1.97)	0.00	1.97 (1.72–2.27)	0.00	2.17 (1.77–2.66)	0.00
Secondary	1.79 (1.49–2.14)	0.00	2.40 (2.05–2.82)	0.00	2.91 (2.35–3.60)	0.00
Higher	5.01 (2.91–8.61)	0.00	9.04 (5.44–15.03)	0.00	12.46 (7.33–21.2)	0.00
**Spouse’s highest education**						
No education	Reference					
Primary	1.94 (1.67–2.25)	0.00	2.25 (1.96–2.57)	0.00	1.71 (1.38–2.13)	0.00
Secondary	1.71 (1.48–1.97)	0.00	2.22 (1.96–2.52)	0.00	2.23 (1.82–2.73)	0.00
Higher	2.89 (2.28–3.65)	0.00	3.67 (2.96–4.54)	0.00	4.38 (3.33–5.77)	0.00
**Who decides respondent’s healthcare**						
Respondent alone	1.28 (1.05–1.56)	0.01	1.25 (1.05–1.49)	0.01	1.84 (1.51–2.25)	0.00
Respondent and Spouse	1.12 (0.99–1.27)	0.08	1.29 (1.15–1.44)	0.00	1.05 (0.92–1.21)	0.45
Spouse alone	Reference					
**Media exposure**						
No exposure	Reference					
Exposed to media	1.29 (1.16–1.43)	0.00	1.48 (1.35–1.63)	0.00	1.73 (1.50-2.00)	0.00
**Ethnicity**						
Hausa/Fulani	Reference					
Yoruba	1.33 (0.90–1.96)	0.15	1.68 (1.22–2.31)	0.00	3.17 (2.22–4.51)	0.00
Igbo	2.55 (1.71–3.83)	0.00	3.16 (2.01–3.88)	0.00	3.82 (2.64–5.52)	0.00
Ibiobio	3.07 (1.55–4.98)	0.00	3.22 (2.09–5.03)	0.00	2.78 (1.28–4.55)	0.00
Others	1.44 (1.25–1.66)	0.00	1.44 (1.26–1.64)	0.00	1.61 (1.29-2.00)	0.00
**Religion**						
Islam	Reference					
Other Christians	0.77 (0.64–0.91)	0.00	0.83 (0.71–0.97)	0.02	0.80 (0.66–0.97)	0.02
Others	1.03 (0.64–1.68)	0.89	0.69 (0.45–1.08)	0.11	0.10 (0.04–0.23)	0.00
**Wealth status**						
Low	Reference					
Middle	1.33 (1.17–1.50)	0.00	1.54 (1.38–1.72)	0.00	1.67 (1.41–1.98)	0.00
Richest	1.58 (1.28–1.95)	0.00	2.11 (1.75–2.54)	0.00	2.29 (1.83–2.87)	0.00
**Children ever-born**						
1 or 2 births	Reference					
3 or 4 births	1.14 (0.98–1.34)	0.10	0.91 (0.79–1.04)	0.17	0.85 (0.72–1.01)	0.07
More than 4 births	1.19 (0.99–1.43)	0.06	0.89 (0.76–1.05)	0.16	0.76 (0.62–0.93)	0.01
**Birth interval**						
First Birth	1.15 (0.94–1.41)	0.17	1.23 (1.03–1.46)	0.02	1.38 (1.11–1.71)	0.00
Less than 36 months	0.91 (0.82–1.01)	0.08	0.87 (0.79–0.95)	0.00	0.92 (0.81–1.04)	0.18
36+	Reference					
**Current employment status**						
Unemployed	Reference					
Employed	0.96 (0.75–1.22)	0.73	0.86 (0.70–1.07)	0.18	0.97 (0.72–1.31)	0.86
**Spouse current employment status**						
Unemployed	Reference					
Employed	1.36 (1.06–1.73)	0.02	1.63 (1.31–2.03)	0.00	1.58 (1.16–2.17)	0.00
**Region**						
North West	Reference					
North central	0.43 (0.37–0.51)	0.00	0.44 (0.38–0.52)	0.00	1.23 (0.96–1.58)	0.11
North East	1.43 (1.26–1.62)	0.00	1.25 (1.11–1.41)	0.00	1.28 (1.01–1.64)	0.04
South East	0.55 (0.34–0.89)	0.01	0.53 (0.35–0.80)	0.00	3.23 (2.03–5.13)	0.00
South South	0.10 (0.08–0.14)	0.00	0.10 (0.08–0.13)	0.00	0.80 (0.58–1.10)	0.17
South West	0.29 (0.20–0.42)	0.00	0.37 (0.27–0.50)	0.00	3.84 (2.69–5.48)	0.00
Residence						
Rural	Reference					
Urban	1.19 (1.04–1.37)	0.01	1.13 (1.01–1.28)	0.04	1.37 (1.19–1.58)	0.00
**Sex of Household head**						
**Male**	Reference					
Female	1.00 (0.81–1.24)	0.97	1.01 (0.85–1.22)	0.88	0.97 (0.79–1.18)	0.74
**Wanted Last child**						
Then	1.07 (0.88–1.31)	0.48	0.84 (0.71–1.01)	0.06	0.84 (0.69–1.03)	0.10
Later	0.78 (0.58–1.04)	0.09	0.7 (0.55–0.90)	0.00	0.63 (0.48–0.84)	0.00
Never	Reference					
**Family Mobility**						
More stable ( > = 5 years)	Reference					
Less stable 0–4 years	1.14 (0.98–1.33)	0.08	1.02 (0.89–1.17)	0.74	1.03 (0.88–1.21)	0.68
**Have health Insurance**						
**No**	Reference					
Yes	0.86 (0.53–1.39)	0.53	1.10 (0.72–1.68)	0.67	1.61 (1.02–2.56)	0.04
**Wife Beating acceptable**						
Yes	Reference					
No	1.21 (1.10–1.33)	0.00	1.40 (1.29–1.53)	0.00	1.71 (1.50–1.95)	0.00
**Community SES Disadvantage**						
Lowest	1.50 (1.17–1.93)	0.00	1.89 (1.52–2.36)	0.00	1.89 (1.38–2.57)	0.00
Middle	1.68 (1.46–1.92)	0.00	1.70 (1.50–1.92)	0.00	1.47 (1.17–1.86)	0.00
Highest	Reference					

All the variables in this Table were significant at *p* < 0.20 in the bivariable analysis

## Discussions

The Federal Ministry of Health in Nigeria developed an orientation package for a new ANC model in Nigeria in 2017 as a response to the new 2016 WHO ANC guideline [12, 19]. The Nigeria model marked its transition from a minimum of 4 visits to a minimum of 8 contacts [19]. The model emphasized having contacts rather than visits. ANC providers were trained, orientated and directed to operationalize the model. We examined Nigeria’s compliance with WHO ANC guidelines on the number of ANC contacts using a nationally representative recent 2018 NDHS data of 21,785 women who responded to questions about antenatal care contacts during the pregnancy of their most recent births (within five years before the survey). Our findings indicate that the proportion of pregnant women who met the new WHO recommendation of a minimum of 8 ANC contacts was low at 20 %. In fact, one of every four pregnant women did not make any ANC contact. However, the proportion not making any contact is lower than the 34.5 % found in a 2013 national study by Fagbamigbe et al. [49]. These results are similar to studies in Bangladesh, Ethiopia and India which showed that most pregnant women had three or fewer ANC contacts instead of the eight required by WHO [22, 34]. This finding also corroborates findings of previous studies that many pregnant women in Nigeria underutilize or do not seek ANC for various reasons [23, 26, 27].

Our study also demonstrates the relationship between sociodemographic characteristics of women and the number of ANC contacts. The lowest proportions of the required number of ANC contacts were recorded among younger, illiterate women, women whose health care access decision was the prerogative of their husbands, and Hausa/Fulani women. This is consistent with local literature in Nigeria [49–52]. In particular, the lowest number of ANC contacts was among teenagers. The number of ANC contacts tended to increase with the age of women. This may be due to the stigmatization of teenage mothers, and older women relying on their experience about pregnancy. Thus, the younger the age of pregnant women was, the fewer were the chances of meeting the WHO recommendation of a minimum of 8 ANC contacts. As earlier pointed out by Aliyu et al., teenage pregnancy is associated with challenges to maternal and infant health outcomes as well as social acceptability of the pregnancy [27]. Many teenage pregnant women avoid public engagements until after delivery for fear of the social stigma attached to such pregnancy at an immature stage of life. As a result of this, very few ANC contacts are made by such women, and in the extreme, ANC contacts are avoided throughout the pregnancy. Thus, Aliyu et al. concluded that the age of pregnant women is associated with their health-seeking behaviour [27]. In Uganda, teenage women were found to be more likely to have less than four ANC contacts probably because of lack of resources and stigmatization [21]. However, in India, both teenage and older women (> 40 years) were more likely to have lower than the required eight contacts than women between these ages [30, 31].

The prevailing low proportion of required ANC contacts was commonest among women from households in the poorest wealth quintiles, women without media exposure, unemployed women, and women who resided in rural areas. Our findings affirmed the findings of previous studies that women with these sociodemographic characteristics have low ANC attendance in Nigeria [23, 53–55]. Women from households in the richest wealth quintile had the highest proportion of women who met the standard number of eight ANC contacts, compared to women in other wealth classes. These findings are in agreement with research findings in Uganda and Bangladesh [21, 32].

The lower level of educational attainment was associated with lower chances of meeting the minimum standard of eight ANC contacts recommended by WHO in this study. We found that pregnant women with higher education were twelve times more likely to achieve the recommended eight ANC contacts than women with no education. Similar findings have been reported in the literature to be associated with higher numbers of ANC contacts [2, 51, 52, 56, 57]. The differences in meeting the WHO recommended standard of a minimum of 8 contacts reflected the wide gap in educational attainment among the women. In addition, spousal educational attainment was associated with a higher number of ANC contacts. The importance of education in achieving the recommended ANC contact cannot be overemphasized. In Bangladesh, higher educational attainment by parents was found to be positively associated with having eight or more ANC contacts [32]. This finding corroborates findings elsewhere that show a positive correlation between a spouse’s level of education and the number of ANC contacts [32, 45]. A higher level of  education among pregnant women and a higher level of education among their spouses may have a positive influence on joint decision making among couples regarding health-seeking during pregnancy. This study also showed that the likelihood of having the required  minimum number of ANC contacts was higher among women who solely or jointly make decisions on healthcare utilization than when such decisions were made by their spouses alone.

Ethnic group is an important factor for ANC attendance and the number of contacts made. Similar to some previous reports, we found that Hausa/Fulani women and other ethnic groups were less likely to meet the required minimum of 8 ANC contacts compared to Yoruba and Igbo/Ibiobio women [23, 26, 36]. Ethnicity may affect norms, and values placed on the use of ANC and the number of ANC contacts thereof. Doctor et al. had reported a low level of education, poor decision making power and early marriage as factors associated with ANC utilization in Hausa/Fulani dominated Northern Nigeria [50]. However, it is not clear whether these ethnic differences are merely a reflection of regional disparities in access to health care. Intra region analysis of ethnic differences in ANC utilization may shed light on this. Similarly, women who were adherents of Islam and other religious faiths were also less likely to meet the minimum standard number of ANC contacts compared to Christian women. Also, the proportion of currently married women, who met the recommended number of contacts, was relatively higher than the other categories of women.

The proportion of women who had the recommended eight ANC contacts was lowest among women that had five or more births. The adjusted relative risk of having 8 or more ANC contacts was 24 % lower among women who had five or more births than those that had only one or two children. These results are similar to findings in Bangladesh [32], India [58] and Ethiopian studies [31]. This is similar to the findings of a study that the higher the parity of a woman the lower the likelihood of an adequate number of ANC contacts in Nigeria [14]. The adjusted risk of having standard 8 ANC contacts was higher among the first births than those whose preceding birth interval was 36 months or longer. This was in agreement with a study in Colombia which reported that women who had birth intervals of less than two years were less likely to have four or more ANC contacts during their pregnancy [33].

A higher proportion of currently employed women met the eight recommended contacts than unemployed women. Similarly, achieving eight ANC contacts was higher among women whose husbands were currently employed. An early study had also asserted that women in clerical and professional jobs were more likely to have four or more ANC contacts [33]. However, in our study, respondents’ current employment status was not significant to making a minimum of 8 contacts but spouses’ employment status was. Other factors associated with making 8 or more contacts were having health insurance, media exposure, acceptability of wife-beating, and the general level of education, employment, media access, and poverty in the community in which the woman resided. Making the minimum required number of ANC contacts was higher among urban women. This could be attributed to closer proximity of ANC providers in urban areas than in rural areas [59]. Our finding is supported by an Uganda study that showed that rural women were less likely to have the 8 recommended ANC contacts than urban women [21].

Furthermore, we found wide variations in the number of ANC contacts across the states in Nigeria. While some states such as Osun and Lagos state had over 70 % adherence to a minimum of 8 ANC contacts, Niger, Gombe, Borno, Yobe, Zamfara and Kebbi States achieved less than 2 %. Also, 23 of the 37 states in Nigeria had over 15 % (the global average) of pregnant women with no ANC contact. It appears these States contribute greatly to the global average. The global average of no ANC contact during pregnancy could fall drastically if the new minimum standards are embraced in these states. Our study shows the regional variation in the number of ANC attendance with North East and North West lagging behind all other regions in Nigeria. The recommended eight ANC contacts were mostly complied with by more than half of the women in the South West. Compliance with the eight recommended ANC contacts was more than 40 % in the South-East and South-South regions. Generally, this study shows that all the northern states recorded a lower proportion of women who achieved the recommended eight ANC contacts compared to the southern states. The findings indicate that regional differences in ANC utilization may be a reflection of educational differences and other community-level factors across the regions. Besides, these factors, the role of instability and conflict in these regional differences needs to be studied. Also, a qualitative study among ANC providers at government and health facilities using in-depth interview and/or focus group discussions will help understand why enough ANC contacts are not made in Nigeria.

## Study strength and limitation

The data was based mostly on respondents’ ability to recall the number of ANC contacts during pregnancy, except for some cases found on ANC cards. Recall bias was not unlikely. We considered this as a limitation. The identified factors are only associated with the number of ANC contacts and should not be taken for causes of the number of contacts as the data used was only cross-sectional in design. More so, the secondary nature of the data, limited choice of explanatory variables. A qualitative study among ANC providers at government and health facility level is needed to support the findings. We did not use multilevel analysis as a multi-level multinomial regression will reduce readability. However, our findings are generalizable as the sample was nationally representative of the Nigerian population. More so, the data source has been reported to use rigorously tested collection tools and procedures and trained personnel on questionnaire administration. Our study is novel in providing local (state) comparison of adherences of WHO guidelines. Most studies on ANC contacts in Nigeria were hitherto restricted to national and regional levels. However, national and regional analysis conceals the state-level scenarios. A major strength of our study is that, to the best of our knowledge, it is the first population-based study in Nigeria to assess adherence to the old WHO-recommended minimum of 4 contacts and a minimum of 8 ANC contacts in Nigeria. It serves as a bridge between past studies on ANC contacts and those that will follow thereafter.

## Conclusions

Despite the newly introduced minimum of ANC contacts by the WHO, a quarter of pregnant women in Nigeria do not make any contact with ANC providers. Nearly three-fifths of pregnant women met the old guideline on a minimum of 4 contacts while only one fifth met the recommended minimum of 8 contacts with ANC providers. Compared with existing local studies on the number of ANC contacts, we found an increase in proportion with at least one ANC contact and at least 4 contacts but none to compare with 8 or more contacts. The increment is a sign of progress but more has to be done for the SDG-3 to be achieved in Nigeria. Nigeria remains far from achieving the targets of the SDG on child and maternal health. The factors associated with the likelihood of meeting the guideline ranged from the wealth quintile of households of the women, their age, religion, education, ethnicity, decision making power, having health insurance, media exposure, acceptability of wife-beating, and how disadvantaged the community was, where a woman resided. This study provided information on where each State stands as far as adherence to the WHO guideline on the minimum number of ANC contacts is concerned, and should guide state-focussed interventions.

## Recommendations

The maternal and child health programmers in Nigeria should review existing policies and develop new policies to adopt, implement and tackle the challenges of adherence to the recommended minimum of 8 contacts with ANC providers. The policy should focus on the removal of barriers that have hitherto limited the number of contacts made. There is a  need to incorporate universal health insurance and a basic health care fund into such policies. All stakeholders including government, non-governmental organizations and community-based organizations including community and religious leaders should harmonize efforts. On the individual levels, women's education, socioeconomic status, adequate education of families, and enhancing decision making power of the women should be a priority. The low level of adherence to the WHO guideline may also be improved upon if the spouses and community are motivated to support and ensure pregnant women are enrolled in the ANC continuum of care. There is a need for urgent interventions to narrow the identified inequalities and substantial disparities in rural-urban regions and states. We recommend qualitative studies to further dissect the reasons why ANC contacts with providers are not made by pregnant women and to also explore the huge variabilities noted in certain states, especially in the Northern part of Nigeria.

## Data Availability

The anonymized data is available in the public domain. The data supporting this article is available on request at www.dhsprogram.com. Extra data is available by emailing Bridgette Wellington (thedhsprogram@gmail.com) the Data Archivist.

## Abbreviations

aRRR: Adjusted relative-risk ratio
ANC: Antenatal care
CI: Confidence Interval
DFF: Decentralized facility financing
EAs: Enumeration areas
FCT: Federal Capital Territory
IRB: Institutional Review Board
IPTp: Intermittent preventive treatment
LGAs: Local government areas
LMIC: Low and Middle-Income Countries
NDHS: Nigeria Demographic Health Survey
NSHIP: Nigerian State Health Investment Programme
PBF: Performance-based financing
PRMM: Pregnancy-related maternal mortality
PSU: Primary sampling unit
SES: Socioeconomic
SSA: Sub-Sahara Africa
SDG: Sustainable development goals
WHO: World Health Organization
UNICEF: United Nations Children’s Fund
UNFPA: United Nations Population Fund

## Declarations

All methods were carried out in accordance with relevant guidelines and regulations. The authors thank the ICF for granting access to the dataset.
